# Elliptical defects create a more adverse biomechanical environment than circular defects in osteochondral lesion of the talus: a finite element analysis

**DOI:** 10.3389/fbioe.2026.1865662

**Published:** 2026-06-23

**Authors:** Zheng Li, Shihang Cao, Xiaocong Liu, Jing Huang, Bozhi Zhao, Taotao Ren, Jun Lu, Junkui Xu

**Affiliations:** 1 Department of Knee Joint Surgery, Honghui Hospital, Xi’an Jiaotong University, Xi’an, Shaanxi, China; 2 Department of Foot and Ankle Surgery (Comprehensive Ward), Honghui Hospital, Xi’an Jiaotong University, Xi’an, Shaanxi, China

**Keywords:** biomechanics, defect shape, finite element analysis, osteochondral lesion of the talus, stress distribution

## Abstract

**Introduction:**

Osteochondral lesion of the talus (OLT) is a common ankle injury that may disturb the biomechanical environment and contribute to progressive articular degeneration. However, in clinical practice, OLT is typically elliptical, whereas most biomechanical studies modeled OLTs as circular defects. This study aimed to compare circular and elliptical OLT defects of different sizes and locations using finite element analysis.

**Methods:**

A subject-specific ankle finite element model was reconstructed from CT data. Full-thickness circular and elliptical defects ranging from 25 to 200 mm^2^ were created in zones 4 and 6 and analyzed under three gait phases. Elliptical defects were defined with an anteroposterior-to-mediolateral axis ratio of 1.5:1. Peak von Mises stress, peak stress location, and regional stress distribution were analyzed.

**Results:**

Compared with circular defects, elliptical defects generally produced higher peak stress and a more adverse redistribution pattern. In zone 4, differences between shapes became more evident in larger defects, and at 200 mm^2^, peak stress in circular defects was 3.77, 4.16, and 5.02 MPa, compared with 4.37, 4.85, and 5.85 MPa in elliptical defects across all gait phases. A similar pattern was observed in zone 6, with elliptical defects also producing higher peak stress than circular defects. These differences were more evident in zone 4 during midstance and push off and were consistently observed in zone 6 across all gait phases. In zone 4, elliptical defects caused the global peak stress to shift from zone 3 to zone 1 at a smaller defect size (150 vs. 175 mm^2^). Elliptical defects also showed a smaller distance between the local peak stress and defect edge than circular defects (2.10 vs. 2.24 mm). Stress redistribution favored the anterior-posterior direction in zone 4 for both shapes, whereas in zone 6 this directional pattern was less consistent, particularly in elliptical defects.

**Conclusion:**

Defect shape substantially alters the biomechanical environment of OLT. Compared with circular defects, elliptical defects produce greater stress concentration, earlier stress redistribution, and a more unfavorable stress pattern, especially in larger lesions. These findings suggest that defect shape should be considered in biomechanical assessment and clinical evaluation.

## 1 Introduction

Osteochondral lesion of the talus (OLT) is a common sports-related injury of the ankle joint (Tomonaga et al., 2024; Nguyen et al., 2024). The incidence of OLT after ankle sprain has been reported to be approximately 6.5% (Zwingmann et al., 2012). If not appropriately managed, these lesions may lead to chronic pain, swelling, restricted range of motion, and progressive degenerative changes (Lan et al., 2021; Rungprai et al., 2017). Moreover, as lesion size and depth progressively increase, the risk of secondary osteoarthritis rises, which can ultimately and substantially compromise patients’ long-term function and quality of life (Mann and Nery, 2024).

Biomechanical factors are considered to play an important role in the development of OLT (Yiğit et al., 2025; Hunt et al., 2012). After injury, the ability of the talar cartilage to absorb and dissipate impact forces is reduced, resulting in uneven stress distribution, localized stress concentration, and abnormal stress transmission (Behling et al., 2024; Wang et al., 2026). Cadaveric ankle experiments have shown that a 15-mm-diameter defect reduces tibiotalar contact area by 20%, increases peak contact stress by 40%, and causes a shift in the location of the most highly loaded region (Anderson et al., 2010). In addition, an abnormal biomechanical environment may further damage the cartilage and subchondral bone at the edge of the defect (Dilley et al., 2023; Guettler et al., 2004). Such mechanical abnormalities may impair chondrocyte homeostasis and extracellular matrix integrity, thereby promoting progressive cartilage degeneration around the defect (Gratz et al., 2009). Therefore, biomechanical analysis is of paramount importance for understanding the mechanisms underlying the progression of lesion-related changes in OLT and for guiding treatment strategies (Fansa et al., 2011; Zhou et al., 2025).

Previous biomechanical studies have demonstrated that lesion size, depth, and location can substantially affect the biomechanical behavior of the ankle in OLT (Li et al., 2022; Li et al., 2020; Aslan et al., 2025). Recent cadaveric studies further suggest that the effect of defect area on cartilage stress may be non-linear, with peak cartilage stress increasing again after the defect exceeds a critical threshold of approximately 120 mm^2^ (Wang et al., 2026). Another study found that increasing defect depth leads to a more unfavorable mechanical environment, particularly when the defect extends beyond 3 mm and involves the subchondral bone (Li et al., 2020). In addition, articular cartilage exhibits location-specific contact patterns during motion, and therefore defects of the same size may lead to different mechanical consequences (Wan et al., 2006). Using the 9-zone grid system of the talar dome, finite element analysis further showed that the most mechanically critical regions varied across gait-related positions, with zone 3 being most critical in dorsiflexion, zone 7 in neutral, and zone 8 in plantarflexion (Aslan et al., 2025). In summary, these findings underscore the biomechanical importance of lesion characteristics in OLT.

However, despite these advances, current biomechanical studies on OLT still have several important limitations. Importantly, OLT morphology in clinical practice is more often elongated or elliptical than circular. Yasui et al. reported mean MRI diameters of 8.0 ± 3.6 mm in the sagittal plane versus 6.1 ± 2.6 mm in the coronal plane, while Deng et al. reported a mean lesion length of 13.38 ± 4.23 mm and width of 9.28 ± 2.28 mm (Yasui et al., 2019; Deng et al., 2020). Therefore, the widespread use of circular defects in biomechanical models represents a mismatch with the shape encountered in clinical practice (Aslan et al., 2025). Because defect geometry is not mechanically neutral, variations in overall shape and edge curvature can alter local load transfer and promote stress concentration around the defect (Peña et al., 2007). This effect may become more pronounced as the defect size increases, because the load-bearing cartilage surrounding the lesion gradually decreases, and the defect margin plays a greater role in stress redistribution. Although lesion size, depth, and location have been widely studied, the independent biomechanical significance of defect shape remains poorly understood. Therefore, a clinically relevant unanswered question is whether elliptical defects, as a more clinically realistic morphology, exhibit different biomechanical behavior from circular defects of the same size.

In this study, we aimed to biomechanically assess circular and elliptical OLT defects of different sizes located in zones 4 and 6 of the talar dome using finite element analysis. We hypothesized that (1) elliptical defects would produce greater stress concentration, and (2) these shape-related differences would become more evident as defect size increased and would vary between defect locations.

## 2 Materials and methods

### 2.1 Overview

This study was approved by the Ethics Committee of Honghui Hospital, Xi’an Jiaotong University (2026-KY-030-01) and conducted in accordance with the local legislation and institutional requirements. The participant provided written informed consent to participate in this study. A healthy adult volunteer was recruited for medical imaging of the ankle joint using computed tomography (CT) scans (256-slice spiral CT, 0.625 mm slice thickness; Siemens, Omaha, NE, United States) for subsequent finite element analysis (FEA). To investigate the biomechanical impact of OLT defect shape, circular and elliptical defects were created in zones 4 and 6 of the talar dome with 8 defect sizes during three key gait phases. Overall, 96 finite element models were created (2 shapes × 8 defect sizes × 2 defect locations × 3 gait phases).

### 2.2 Three-dimensional modeling

**FIGURE 1:**
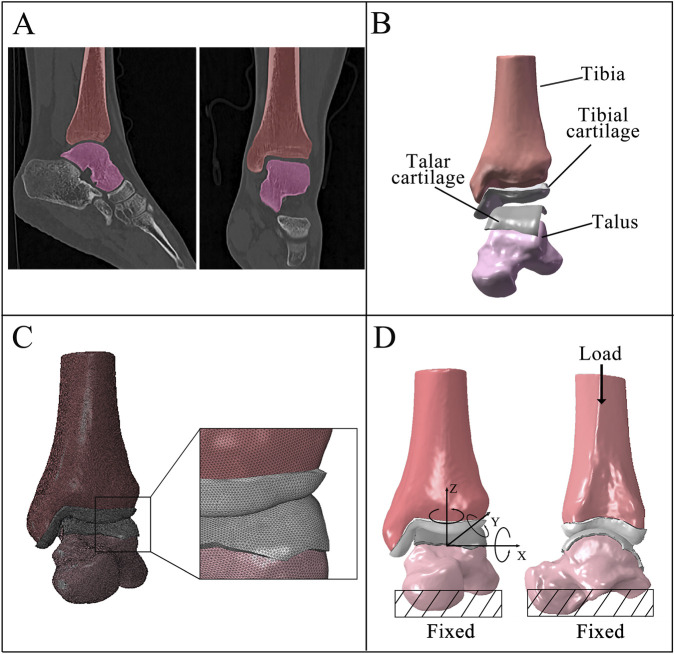
Finite element modeling workflow. **(A)** Segmentation of the tibia and talus in CT images; **(B)** Cartilage modeling of the tibiotalar joint; **(C)** Finite element mesh of the model consisting of tetrahedral elements; **(D)** Loading and boundary conditions.

**FIGURE 2:**
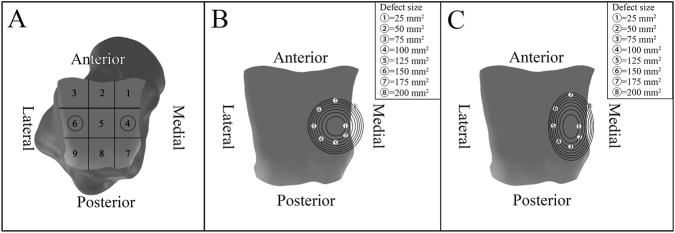
Classification of different cartilage defects. **(A)** Defects were created in zones 4 and 6 using the nine-zone grid method; **(B)** Circular defects with areas ranging from 25 to 200 mm^2^; **(C)** Elliptical defects with areas ranging from 25 to 200 mm^2^.

The workflow of the finite element modeling is shown in Figure 1. A preliminary three-dimensional model comprising the tibia and talus was reconstructed using CT scans in Mimics (Materialise, Belgium) (Figure 1A), and the bones were smoothed in Geomagic Wrap (Geomagic, Durham, NC, United States). The articular cartilage was modeled in SolidWorks (Dassault Systèmes, France) by offsetting tibial and talar articular surfaces with a uniform thickness of 1.5 mm (Figure 1B). Subsequently, the defect models were created in zones 4 and 6 according to the nine-zone grid method, representing common clinical injuries with full-thickness defects (Raikin et al., 2007; Orr et al., 2012). Additionally, defect sizes ranging from 25 to 200 mm^2^ (25, 50, 75, 100, 125, 150, 175, and 200 mm^2^) were modeled as either circular or elliptical, with the elliptical defects defined by an anteroposterior-to-mediolateral axis ratio of 1.5:1 (Figure 2).

### 2.3 Finite element modeling technique

Tetrahedral elements were generated in HyperMesh (Altair, Troy, MI, United States) and mesh sensitivity analysis was conducted in Abaqus 2021 (Dassault Systèmes Simulia, Paris, France) with element sizes of 0.25, 0.50, 0.75 and 1.00 mm. The element size was set to 0.5 mm based on the sensitivity analysis (Supplementary Material S1). The material properties assigned to bone and cartilage are summarized in Table 1 (Ruan et al., 2023). A frictionless contact interaction was defined between the two cartilage surfaces, and the contact between cartilage and bone was defined as “tie”. Based on previous literature, boundary and loading conditions corresponding to the three gait phases (impact, midstance, push off) were applied (Giddings et al., 2000). Resultant forces and moments were applied to a reference point located at the center of rotation of the ankle, which was kinematically coupled to the proximal tibia. The relative tibiotalar position was kept constant across gait phases. The inferior surface of the talus was fully fixed in six degrees of freedom (Figure 1D).

**TABLE 1:** Material properties of bone and cartilage.

Material	Young’s modulus (MPa)	Poisson’s ratio
Bone	7,300	0.3
Cartilage	Hyperelastic (ogden) (μ = 2.43, a = 12.45, D = 0.176)	

### 2.4 Model validation

The subject-specific model in the neutral position was compared with the experimental data reported by Anderson et al. (2007) for model validation. To ensure consistency, identical boundary conditions and load magnitudes were applied to the model of this study. The simulation results were consistent with previously published data (Supplementary Material S2).

### 2.5 Data analysis

**FIGURE 3:**
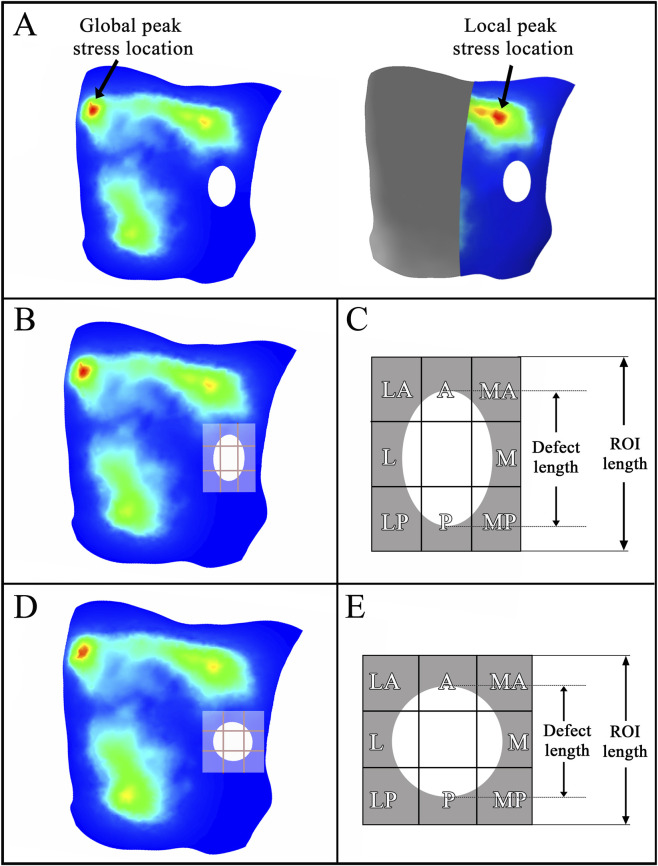
Definition of stress measurement regions. **(A)** The distance from the global and local peak stress location to the defect edge; **(B,C)** The region of interest extending 1.5 mm around the elliptical defect; **(D,E)** The region of interest extending 1.5 mm around the circular defect.

The primary evaluation metric in this study was the von Mises stress. The peak von Mises stresses in the global and local regions were recorded, where the local region was defined as half of the total cartilage on the defect side (Figure 3A). To further investigate stress distribution in the area surrounding the defect, a rectangular region of interest (ROI) centered on the defect was defined. The ROI extended 1.5 mm beyond the defect edge in all directions. For circular defects, the ROI was a square with a side length equal to the defect diameter plus 3 mm. For elliptical defects, the ROI was a rectangle aligned with the anteroposterior and mediolateral axes, with side lengths equal to the defect anteroposterior length plus 3 mm and mediolateral width plus 3 mm, respectively. Furthermore, the ROI was divided into nine grids. The three grids covering the medial cartilage were grouped as zone M, whereas the remaining grids corresponded to the lateral (L), anterior (A), and posterior (P) cartilage regions, respectively (Figures 3B–E). The average stress in each zone was calculated from the nodal stress values within that zone. All metrics were extracted and processed using Python 3.12 with custom scripts. The results were compared descriptively between circular and elliptical defects under matched defect size, location, and gait phase conditions.

## 3 Results

### 3.1 Peak von mises stress between circular and elliptical defects

**FIGURE 4:**
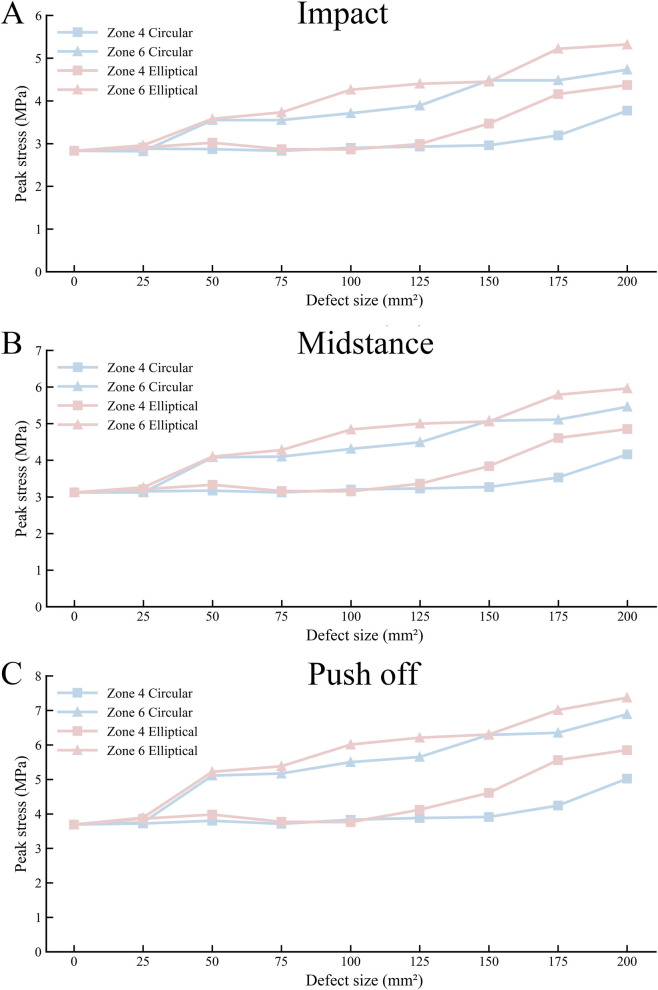
Peak von Mises stress across defect sizes under three gait phases **(A)** Impact; **(B)** Midstance; **(C)** Push off. Different lines represent circular and elliptical defects in zones 4 and 6. A defect size of 0 mm^2^ represents the intact condition.

**FIGURE 5:**
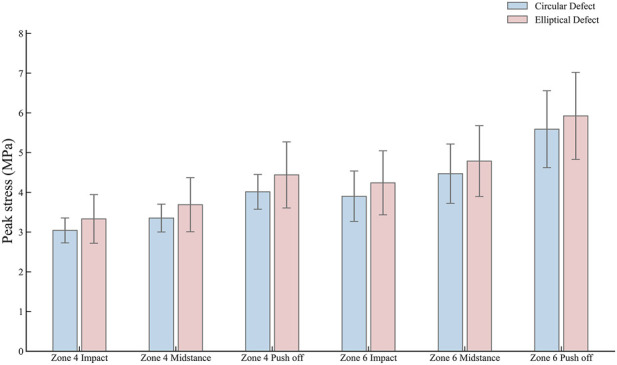
Bar graphs showing peak von Mises stress in circular and elliptical defects across gait phases and defect locations. Bars show mean values with error bars indicating standard deviation.

The peak von Mises stress across different defect sizes at various gait phases is presented in Figure 4, and the corresponding peak stress values are shown in Tables 2, 3. In the intact condition, the peak von Mises stress was 2.83 MPa during impact, 3.14 MPa during midstance, and 3.69 MPa during push off. Peak von Mises stress increased progressively with defect size in both zones, but the magnitude and pattern of this increase differed between shapes. In zone 4, peak stress remained close to the intact condition for defect sizes of 25–125 mm^2^. In circular defects, the values ranged from 2.83 to 2.93 MPa during impact, 3.12–3.23 MPa during midstance, and 3.71–3.88 MPa during push off. In elliptical defects, the corresponding values were 2.86–3.02 MPa, 3.15–3.36 MPa, and 3.76–4.12 MPa. However, from 150 mm^2^ onward, peak stress increased more markedly, particularly in elliptical defects, reaching 4.37 MPa during impact, 4.85 MPa during midstance, and 5.85 MPa during push off at 200 mm^2^. In zone 6, peak stress remained close to the intact condition at 25 mm^2^ and increased more clearly from 50 mm^2^ onward. In circular defects, peak stress increased progressively from 3.55 to 4.73 MPa during impact, from 4.08 to 5.46 MPa during midstance, and from 5.11 to 6.89 MPa during push off as defect size increased from 50 to 200 mm^2^. In elliptical defects, the corresponding values increased from 3.58 to 5.32 MPa, 4.10–5.96 MPa, and 5.22–7.37 MPa. Descriptive comparisons between matched circular and elliptical defects are shown in Figure 5. Elliptical defects produced higher peak stress than circular defects in most conditions.

**TABLE 2:** Peak von Mises stress (MPa) during different gait phases in zone 4.

Size (mm^2^)	Circular			Elliptical		
	Impact	Midstance	Push off	Impact	Midstance	Push off
25	2.88	3.15	3.72	2.91	3.21	3.86
50	2.87	3.17	3.80	3.02	3.33	3.98
75	2.83	3.12	3.71	2.87	3.16	3.77
100	2.90	3.20	3.83	2.86	3.15	3.76
125	2.93	3.23	3.88	2.99	3.36	4.12
150	2.96	3.27	3.91	3.47	3.84	4.61
175	3.19	3.53	4.24	4.16	4.61	5.56
200	3.77	4.16	5.02	4.37	4.85	5.85

**TABLE 3:** Peak von Mises stress (MPa) during different gait phases in zone 6.

Size (mm^2^)	Circular			Elliptical		
	Impact	Midstance	Push off	Impact	Midstance	Push off
25	2.82	3.12	3.75	2.96	3.26	3.89
50	3.55	4.08	5.11	3.58	4.10	5.22
75	3.55	4.10	5.17	3.73	4.28	5.38
100	3.71	4.31	5.50	4.26	4.84	6.01
125	3.89	4.49	5.65	4.40	5.00	6.21
150	4.48	5.08	6.29	4.45	5.06	6.30
175	4.48	5.11	6.35	5.22	5.79	7.01
200	4.73	5.46	6.89	5.32	5.96	7.37

### 3.2 Spatial distribution characteristics of peak von mises stress

**FIGURE 6:**
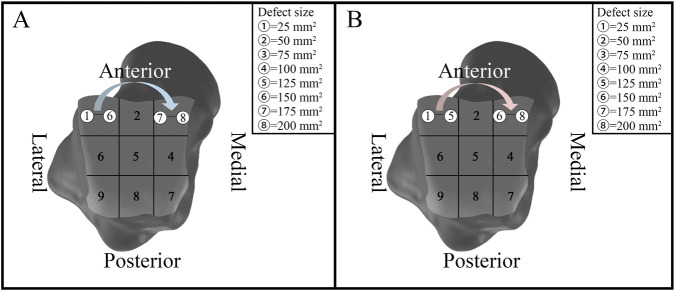
Global peak stress locations for defects located in zone 4. Numbers 1-8 represent defect sizes ranging from 25 to 200 mm^2^. **(A)** For circular defects, the global peak stress remained in zone 3 up to 150 mm^2^ and shifted to zone 1 at 175 and 200 mm^2^; **(B)** For elliptical defects, the global peak stress remained in zone 3 up to 125 mm^2^ and shifted to zone 1 at 150 mm^2^ and larger.

The stress distribution maps for circular and elliptical defects under all conditions are provided in Supplementary Material S3. When the defect was located in zone 6, global and local peak stress were located in zone 3 for a defect size of 25 mm^2^, but shifted to the defect edge for all larger defect sizes. Additionally, Table 4 shows the distance from the local peak stress to the defect edge in the local zone when the defect was located in zone 4. In both circular and elliptical defects, the distance generally decreased as defect size increased. In circular defects, the minimum distance was 2.24 mm, occurring at 150 mm^2^ during all gait phases, whereas in elliptical defects a smaller minimum distance of 2.10 mm was observed earlier at 100 mm^2^ during push off. When the defect size was less than or equal to 125 mm^2^, the distance from the local peak stress to the defect edge was generally shorter for elliptical defects than for circular defects. Additionally, the global peak stress location varied with both defect shape and defect size (Figure 6). For circular defects, global peak stress remained in zone 3 at defect sizes up to 150 mm^2^ and shifted to zone 1 at 175 and 200 mm^2^. In contrast, for elliptical defects, this shift occurred earlier, with peak stress remaining in zone 3 up to 125 mm^2^ and shifting to zone 1 at 150 mm^2^ and larger.

**TABLE 4:** Distance (mm) from local peak stress location to defect edge for circular and elliptical defects in zone 4.

Size (mm^2^)	Circular			Elliptical		
	Impact	Midstance	Push off	Impact	Midstance	Push off
25	8.34	8.34	8.31	7.90	7.90	7.90
50	7.07	7.09	7.09	6.57	6.57	6.57
75	6.54	6.54	6.19	5.40	5.40	5.40
100	5.49	5.49	5.49	4.93	4.93	2.10
125	4.45	4.45	4.45	2.24	2.24	2.24
150	2.24	2.24	2.24	2.77	2.77	2.46
175	2.34	2.34	2.46	2.88	2.88	2.88
200	2.71	2.71	2.33	2.65	2.65	2.65

### 3.3 Regional differences in mean von mises stress around the defect

Table 5 presents the mean von Mises stress averaged across the three gait phases in the combined anterior and posterior regions (A + P) and the combined medial and lateral regions (M + L) surrounding the defect. In zone 4, the mean stress in A + P was consistently greater than that in M + L for both circular and elliptical defects. In zone 6, circular defects also showed a tendency toward higher A + P stress than M + L stress, whereas elliptical defects showed a less consistent directional pattern. To further quantify this directional difference in stress redistribution, the difference between the combined anterior-posterior stress and the combined medial-lateral stress (AP-ML stress difference) was examined across defect sizes in Table 6. In zone 4, AP-ML stress difference increased overall with defect size in circular defects, rising from 0.02 at 25 mm^2^ to 0.81 at 175 mm^2^. In elliptical defects, AP-ML stress difference increased more rapidly at smaller defect sizes, reaching a maximum of 0.86 at 100 mm^2^, and then gradually decreased to 0.31 at 200 mm^2^. In zone 6, no consistent trend in AP-ML stress difference was observed with increasing defect size.

**TABLE 5:** Mean von Mises stress (MPa) averaged across the three gait phases in the combined anterior-posterior (A + P) and medial-lateral (M + L) regions around circular and elliptical defects in zones 4 and 6.

Size (mm^2^)	Zone 4				Zone 6			
	A + P circular	M + L circular	A + P elliptical	M + L elliptical	A + P circular	M + L circular	A + P elliptical	M + L elliptical
25	0.13	0.11	0.18	0.10	1.61	1.39	1.88	1.69
50	0.21	0.14	0.43	0.13	1.79	1.68	1.93	1.82
75	0.42	0.14	0.78	0.15	1.25	1.27	1.51	1.87
100	0.61	0.14	1.04	0.18	1.21	1.06	1.38	1.93
125	0.81	0.14	0.94	0.16	1.45	1.28	1.30	1.69
150	0.79	0.16	0.75	0.17	1.34	1.16	1.42	1.59
175	1.00	0.19	0.67	0.19	1.62	1.06	1.65	1.53
200	0.86	0.21	0.50	0.19	1.53	0.78	1.71	1.40

**TABLE 6:** Difference (MPa) between the combined anterior-posterior stress and the combined medial-lateral stress across defect sizes for circular and elliptical defects in zones 4 and 6.

Size (mm^2^)	Zone 4 circular	Zone 4 elliptical	Zone 6 circular	Zone 6 elliptical
25	0.02	0.08	0.22	0.19
50	0.07	0.30	0.11	0.11
75	0.28	0.63	−0.02	−0.36
100	0.47	0.86	0.15	−0.55
125	0.67	0.78	0.17	−0.39
150	0.63	0.58	0.18	−0.17
175	0.81	0.48	0.56	0.12
200	0.65	0.31	0.75	0.31

## 4 Discussion

The main finding of the present study was that lesion shape had an important influence on the biomechanical environment of OLT. Under matched conditions of defect size and location, elliptical defects generally produced higher peak von Mises stress on the talar cartilage than circular defects. This pattern was reflected not only in the global peak stress of the talar cartilage but also in local stress behavior around the defect. In zone 4, the global peak stress shifted from zone 3 to zone 1 as defect size increased, and this shift occurred at a smaller defect size in elliptical defects than in circular defects. Further analysis of the ROI surrounding the defect suggested a redistribution of stress along the anterior-posterior direction, particularly in zone 4. Overall, these findings highlight the biomechanical importance of lesion shape in OLT.

Clinically, OLT typically exhibits an elongated rather than circular morphology (Yasui et al., 2019; Chen et al., 2023). However, many previous biomechanical studies have modeled OLT defects as regular circular defects, with limited attention to shape as an independent variable (Ruan et al., 2023). Imaging and clinical studies have reported that the anteroposterior dimension is commonly greater than the transverse dimension, with reported ratios approximating 1.3 to 1.5 (Orr et al., 2017; Khan et al., 2024). Therefore, an elliptical defect with an aspect ratio of 1.5:1 was selected in the present study as a reasonable representation. Our study found that, compared with circular defects, elliptical defects produced a different stress environment in the talar cartilage including differences in peak stress magnitude and spatial distribution. Specifically, our results demonstrated that elliptical defects generally produced higher peak von Mises stress than circular defects under otherwise matched conditions. Although previous OLT studies have focused primarily on defect size rather than shape, this pattern is biomechanically plausible given prior evidence that local defect geometry can influence rim mechanics and stress redistribution (Zevenbergen et al., 2018; Yanke et al., 2019). Notably, our results suggest that the influence of defect shape is not limited to stress magnitude, but also extends to the spatial pattern of stress distribution. For defects located in zone 4, the global peak stress location shifted from zone 3 to zone 1 at a smaller defect size in elliptical defects than in circular defects, occurring at 150 mm^2^ rather than 175 mm^2^. This finding may indicate that elliptical geometry is more likely to disturb the original load-bearing pathway and to redirect peak mechanical demand toward adjacent regions at an earlier stage.

Current surgical decision-making for OLT is often guided by lesion size, particularly when choosing between bone marrow stimulation (BMS) and more restorative surgical procedures such as autologous osteochondral transplantation (AOT) (Walther et al., 2024; Ramponi et al., 2017; Flynn et al., 2016). However, reported surgical thresholds vary across studies, probably reflecting differences in study design, clinical endpoints and treatment context. Earlier clinical studies identified 150 mm^2^ as an important cutoff associated with poorer outcomes after BMS (Choi et al., 2009). Later reviews proposed a more conservative threshold of 100 or 107.4 mm^2^, while more recent cadaveric and computational studies suggested a biomechanically relevant threshold for selecting AOT at approximately 120 mm^2^ (Wang et al., 2026; Wang et al., 2021). In our study, peak stress showed a more evident increasing trend after approximately 150 mm^2^ in zone 4, while a similar trend was observed from approximately 50 mm^2^ in zone 6. These findings provide a biomechanical perspective suggesting that lesion size alone may not fully characterize the mechanical environment of OLT. Therefore, defect shape and location may be useful complementary factors in future biomechanical assessment and clinical evaluation, although their role in treatment selection requires further validation with clinical outcome data.

In the progression of OLT, lesion shape is not merely a static geometric descriptor, as alterations in stress environment may also influence its subsequent pattern of development (Yanke et al., 2019). Our study showed that, for smaller zone 4 defects, the local peak stress in elliptical defects tended to occur closer to the defect edge than in circular defects. This pattern is partly consistent with the cadaveric study by Hunt et al., in which increasing OLT size was associated with peak stress shifting closer to the defect edge (Hunt et al., 2012). This difference suggests that an elongated lesion may promote greater edge stress concentration, thereby rendering the cartilage adjacent to the defect edge more susceptible to further damage. In addition, the ROI analysis further demonstrated that the biomechanical effects of the defect extended into the adjacent regions. A previous finite element study showed that even when the initial defect was modeled as circular, the subsequent stress distribution predicted preferential cartilage wear in a more elongated pattern (Ruan et al., 2023). Our results were directionally similar, suggesting that once a defect occurs, the abnormal stress environment may lead to further deterioration of the lesion and promote its progression toward an elongated morphology. Stress difference analysis within the ROI further showed that, in zone 4, mechanical overload was preferentially redistributed around the lesion along the anteroposterior direction. In contrast, the ROI analysis in zone 6 showed a more variable redistribution pattern, particularly in elliptical defects. In moderate-sized elliptical defects, stress tended to redistribute toward the mediolateral regions rather than along the anteroposterior direction. Compared with Zone 4, Zone 6 is located more laterally on the talar dome and is characterized by a smaller lateral curvature radius (Siegler et al., 2014). This geometric and location difference may make local stress redistribution more susceptible to peripheral contact mechanics. In this region, the elongated defect may therefore alter load sharing around the defect edge and redirect stress toward the medial and lateral margins. Therefore, defect shape may be a factor that should be considered in future biomechanical investigations of OLT.

Understanding the biomechanical environment of injury-related changes is important for clarifying the mechanisms underlying lesion progression and for assessing its potential clinical evolution (Mangwani et al., 2024; Ge et al., 2026). In our study, the presence of an OLT increased peak stress in the talar cartilage, with larger defects generally producing greater stress elevation. This is broadly consistent with previous biomechanical studies showing that lesion size substantially alters the stress environment of the ankle joint (Hunt et al., 2012). Li et al. found that increasing defect size progressively impaired ankle stability and that marked stress abnormalities became evident once the lesion reached 6 × 6 mm^2^ (Li et al., 2022). However, the literature is inconsistent regarding the threshold size at which the mechanical environment begins to deteriorate significantly. Recent cadaveric and computational studies have suggested that a new critical change occurs when the defect size reaches 120 mm^2^ (Wang et al., 2026). In our study, we found that more marked biomechanical worsening became evident when the defect size exceeded 150 mm^2^ for lesions located in zone 4 and 50 mm^2^ for lesions located in zone 6. This discrepancy can be partially attributed to differences in the location and shape of the defects. Overall, these results indicate that the biomechanical impact of OLT depends not only on defect size, but also on defect location and shape.

However, this study has several limitations. First, it was based on a simplified finite element model of the ankle joint reconstructed from a single healthy volunteer. Although the major anatomical structures were included, certain soft tissues, cartilage thickness, ligaments and loading conditions were simplified. Our model may not fully capture the potential biomechanical effects of gait-dependent changes in ankle posture on cartilage contact mechanics and stress redistribution. Furthermore, the use of a uniform cartilage thickness may affect the absolute values of local edge stress calculations, particularly in thinner lateral regions such as zone 6, where the stress values might be greater. Second, although the present study provided biomechanical insights based on finite element analysis, the findings were not further validated by cadaveric experiments and clinical research. Finally, this study represents a preliminary exploration of the role of clinically relevant defect shape in OLT. Further studies are needed to investigate a broader range of defect geometries and locations and to compare their mechanical effects.

## 5 Conclusion

In this study, defect shape substantially altered the biomechanical environment of OLT. Under otherwise identical conditions, elliptical defects generally produced higher peak von Mises stress than circular defects and were associated with earlier stress redistribution and stress concentration closer to the defect edge. In addition, the apparent biomechanical transition point varied by lesion location, occurring at approximately 150 mm^2^ in zone 4 and 50 mm^2^ in zone 6. Therefore, a single universal size threshold may be insufficient for surgical decision-making, and defect location and shape should be taken into account in the evaluation and management of OLT.

## Data Availability

The raw data supporting the conclusions of this article will be made available by the authors, without undue reservation.
