# Supplementary material for: Elliptical defects create a more adverse biomechanical environment than circular defects in osteochondral lesion of the talus: a finite element analysis
Source: Front Bioeng Biotechnol. 2026 Jun 23;14:1865662. doi: 10.3389/fbioe.2026.1865662 (PMC13337844; doi:10.3389/fbioe.2026.1865662)
Supplement: Supplementary file 1 [file Supplementaryfile1.docx]

Supplementary Material 1

Mesh sensitivity

A mesh sensitivity analysis was performed using four element sizes: 0.25, 0.5, 0.75, and 1.0 mm. Corresponding peak stresses on the articular surface were 2.88, 2.72, 2.63, and 2.36 MPa, respectively (Figure S1). Mesh convergence was considered achieved at the 0.5 mm element size, where the relative change in peak stress dropped below 5%.


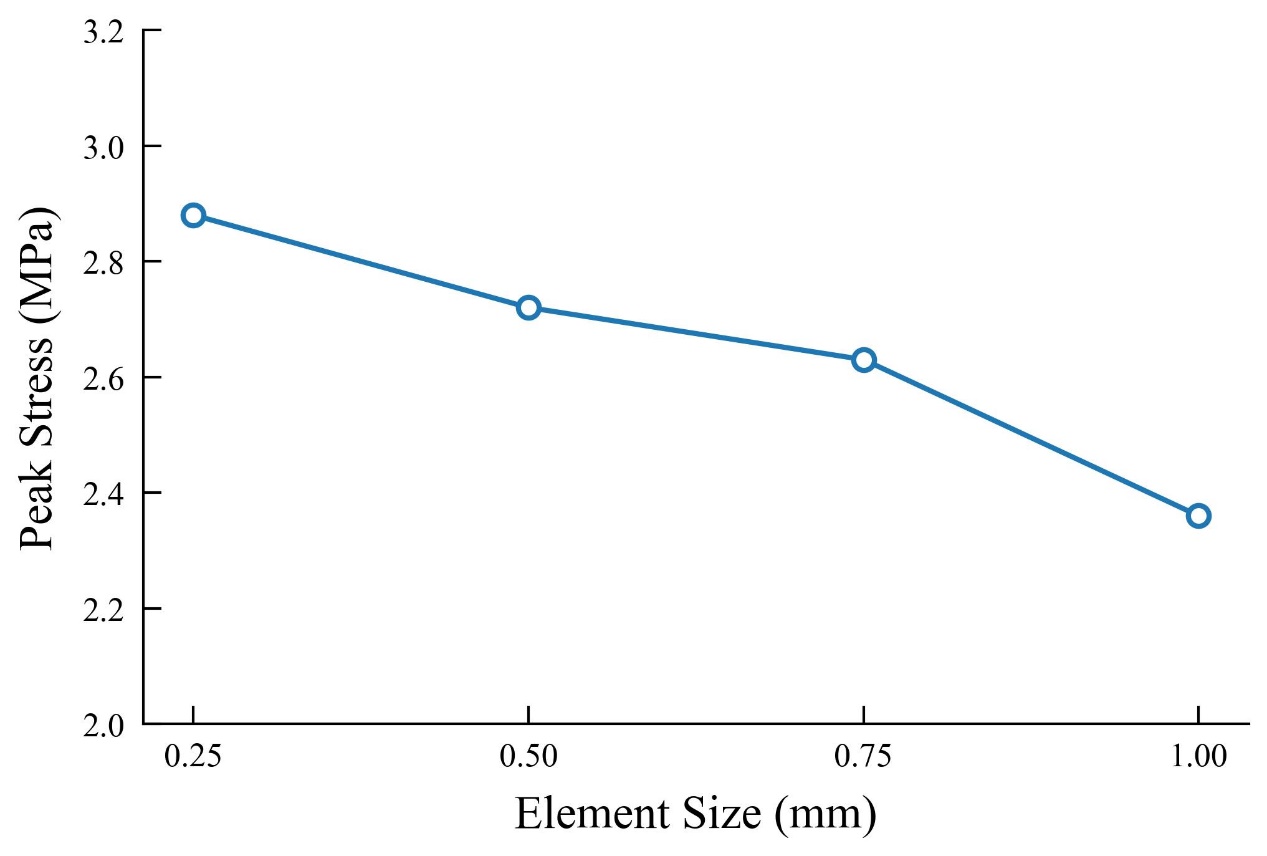


**Figure S1.** Mesh sensitivity analysis of the finite element model.
