# Supplementary material for: Elliptical defects create a more adverse biomechanical environment than circular defects in osteochondral lesion of the talus: a finite element analysis
Source: Front Bioeng Biotechnol. 2026 Jun 23;14:1865662. doi: 10.3389/fbioe.2026.1865662 (PMC13337844; doi:10.3389/fbioe.2026.1865662)
Supplement: Supplementary file 2 [file Supplementaryfile3.docx]

Supplementary Material 3

Von Mises stress distribution plots

This supplementary material presents von Mises stress distribution plots from the subject-specific finite element model, illustrating how the defect alters the biomechanical environment of the talar cartilage in osteochondral lesions of the talus (Figure S1 and S2).





**Figure S1.** Von Mises stress distribution plots during different gait phases in zone 4.

**

**

**Figure S2.** Von Mises stress distribution plots during different gait phases in zone 6.
