# Supplementary material for: Elliptical defects create a more adverse biomechanical environment than circular defects in osteochondral lesion of the talus: a finite element analysis
Source: Front Bioeng Biotechnol. 2026 Jun 23;14:1865662. doi: 10.3389/fbioe.2026.1865662 (PMC13337844; doi:10.3389/fbioe.2026.1865662)
Supplement: Supplementary file 3 [file Supplementaryfile2.docx]

Supplementary Material 2

In this study, the finite element models of native cartilage were validated against the experimental pressure-sensor data reported by Anderson et al.(1), who measured tibiotalar cartilage contact characteristics under a 600 N axial load using pressure sensors. To ensure consistency, we applied the same joint anatomy, loading conditions, boundary constraints, and joint postures to biological finite element models constructed from four representative subjects. The resulting predicted contact patterns and contact pressures demonstrated strong agreement with the in vitro findings (1) (Figure S2, Table S1).


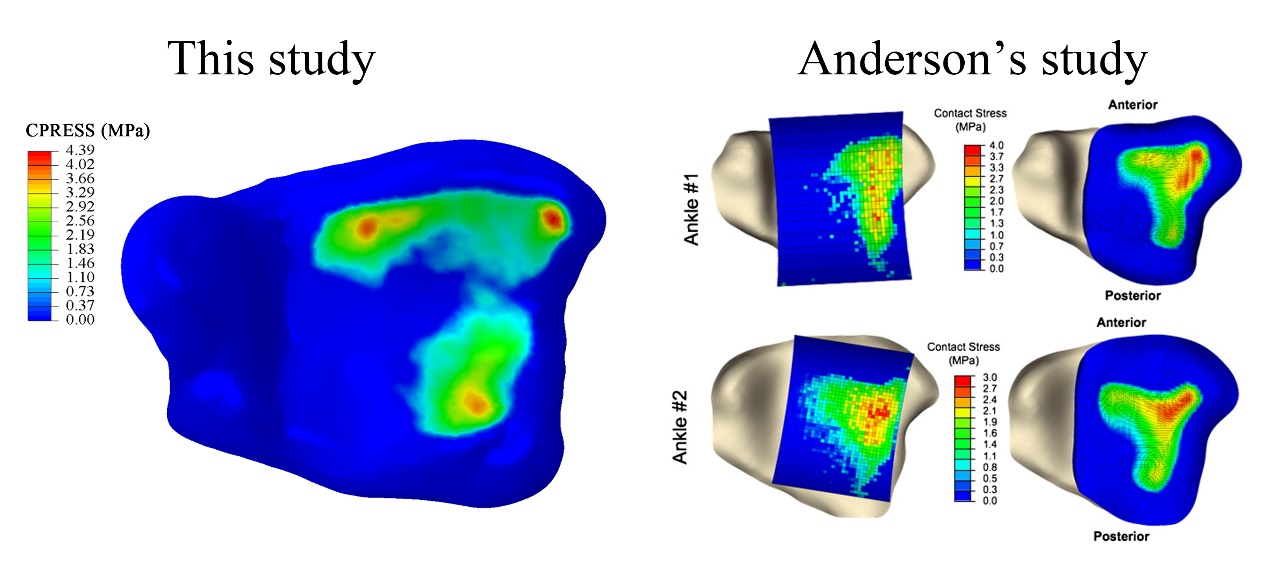


**Figure S2.** Comparison of contact stress distribution between our study’s subject and those reported by Anderson.

**Table S1.** Comparison of peak contact stress values between subjects in our study and those reported by Anderson

|  | This Study | Anderson’s Study | |
| --- | --- | --- | --- |
|  |  | Ankle #1 | Ankle #2 |
| Contact Stress (MPa) | 4.39 | 3.74 | 2.74 |

1. Anderson DD, Goldsworthy JK, Li W, James Rudert M, Tochigi Y, Brown TD. Physical validation of a patient-specific contact finite element model of the ankle. J Biomech. 2007;40(8):1662-9.
